# Supplementary material for: Replication-associated formation and repair of human topoisomerase IIIα cleavage complexes
Source: Nat Commun. 2023 Apr 6;14:1925. doi: 10.1038/s41467-023-37498-6 (PMC10079683; doi:10.1038/s41467-023-37498-6)
Supplement: Supplementary file 1 — Supplementary Information [file 41467_2023_37498_MOESM1_ESM.pdf]

Supplementary Information for

Replication-associated formation and repair of human topoisomerase IIIα cleavage complexes

Liton Kumar Saha, Sourav Saha, Xi Yang, Shar-yin Naomi Huang, Yilun Sun, Ukhyun Jo and Yves Pommier *

* Corresponding Author: Yves Pommier
Email: pommier@nih.gov

This PDF file includes:

Supplementary Figures 1-10

Supplementary Figure Legends

Supplementary Figure 1

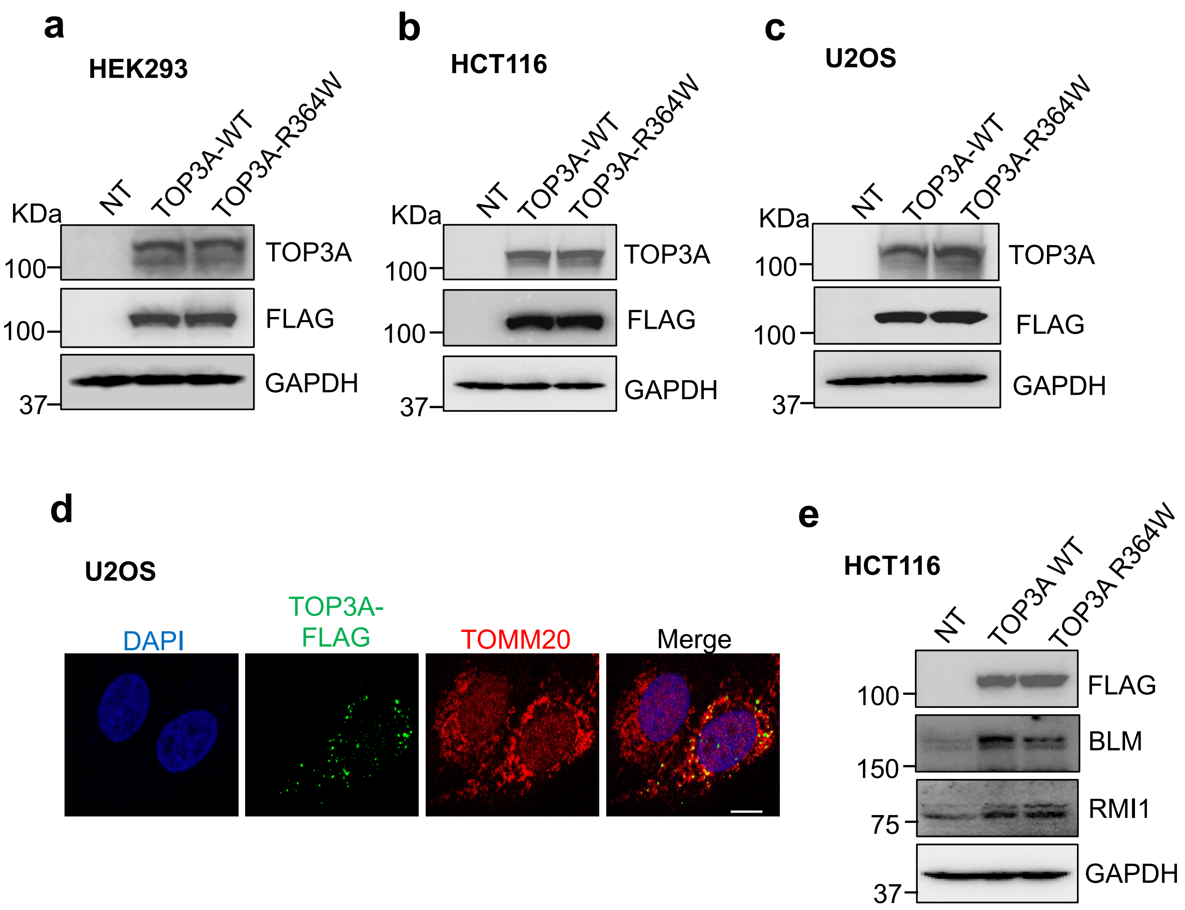

Supplementary Figure 1: Ectopic expression of TOP3A-WT and TOP3A-R364W.

a–c Western blots of cells over-expressing TOP3A-WT and TOP3A-R364W in HEK293 (**a**), HCT116 (**b**) and U2OS cells (**c**). Cells were transfected with the indicated FLAG-tagged TOP3A constructs for 48 h and subjected to Western blotting with the indicated antibodies. GAPDH was used as loading control. **d** Representative confocal microscopy images of U2OS cells showing localization of TOP3A in mitochondria. After fixation, for immunofluorescence, cells were stained with anti- TOMM20 (Red, mitochondria surface marker) and anti-FLAG (Green) primary antibodies. Scale bar: 5μm. **e** Western blot analysis of HCT116 cells after transfection with FLAG-tagged TOP3A WT and TOP3A R364W constructs for 48 h using the indicated antibodies.

Supplementary Figure 2

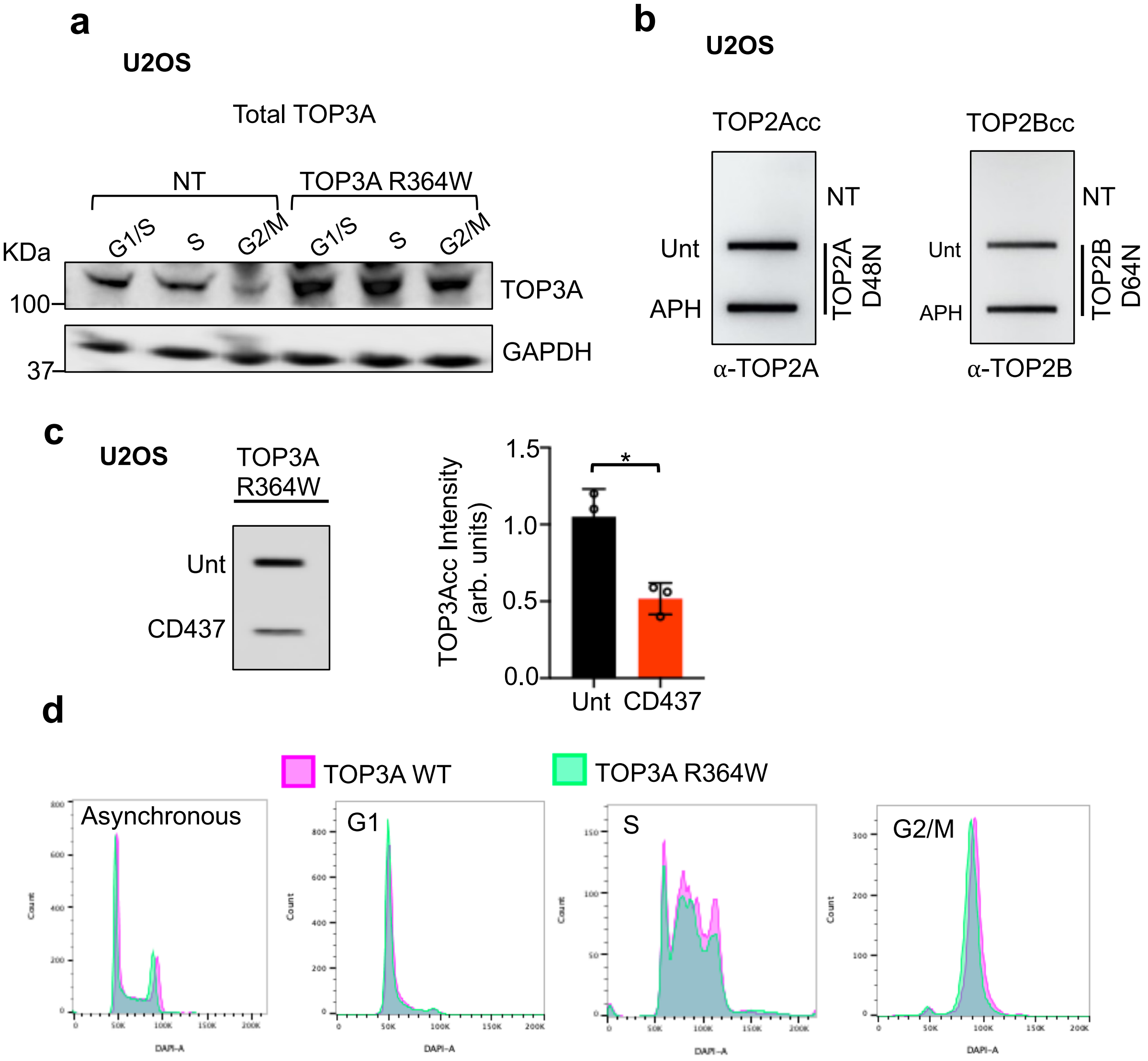

Supplementary Figure 2: Cell cycle phase-dependent expression of TOP3A-R364W and impact of polymerase α inhibition on TOP3Accs.

a Western blots of level of TOP3A in U2OS cells over-expressing TOP3A-R364W and mock-transfected (NT) in different phases of the cell cycle. **b** Aphidicolin treatment causes no reduction in TOP2Accs and TOP2Bccs levels. Representative slot blot for the detection of TOP2Accs and TOP2Bccs by RADAR assay in U2OS cells transfected with the indicated self-trapping TOP2A and TOP2B plasmid constructs respectively for 48 h. TOP2Accs and TOP2Bccs were detected with anti-TOP2A and anti-TOP2B antibody, respectively. **c** CD437 treatment reduces TOP3Accs levels. TOP3A-R364W-transfected U2OS cells were pretreated with 1.5 μM DNA polymerase α inhibitor, CD437, for 1 h before cell harvesting and RADAR assays. Left panel: representative slot blot probed with anti-TOP3A antibody. Right panel: quantitation for 3 independent experiments as shown on the left panel. Error bar indicates mean ± SD. P-values were obtained by two-tailed unpaired *t*-test with Welch's correction. * p=0.0188. **d** TOP3A-WT and TOP3A-R364W-expressing U2OS cells were synchronized in G_1, S and G2/M phases of the cell cycle with double-thymidine block. Histogram shows cell cycle distribution by fluorescence-activated cell sorting (FACS) analysis of cellular DNA content (DAPI).

Supplementary Figure 3

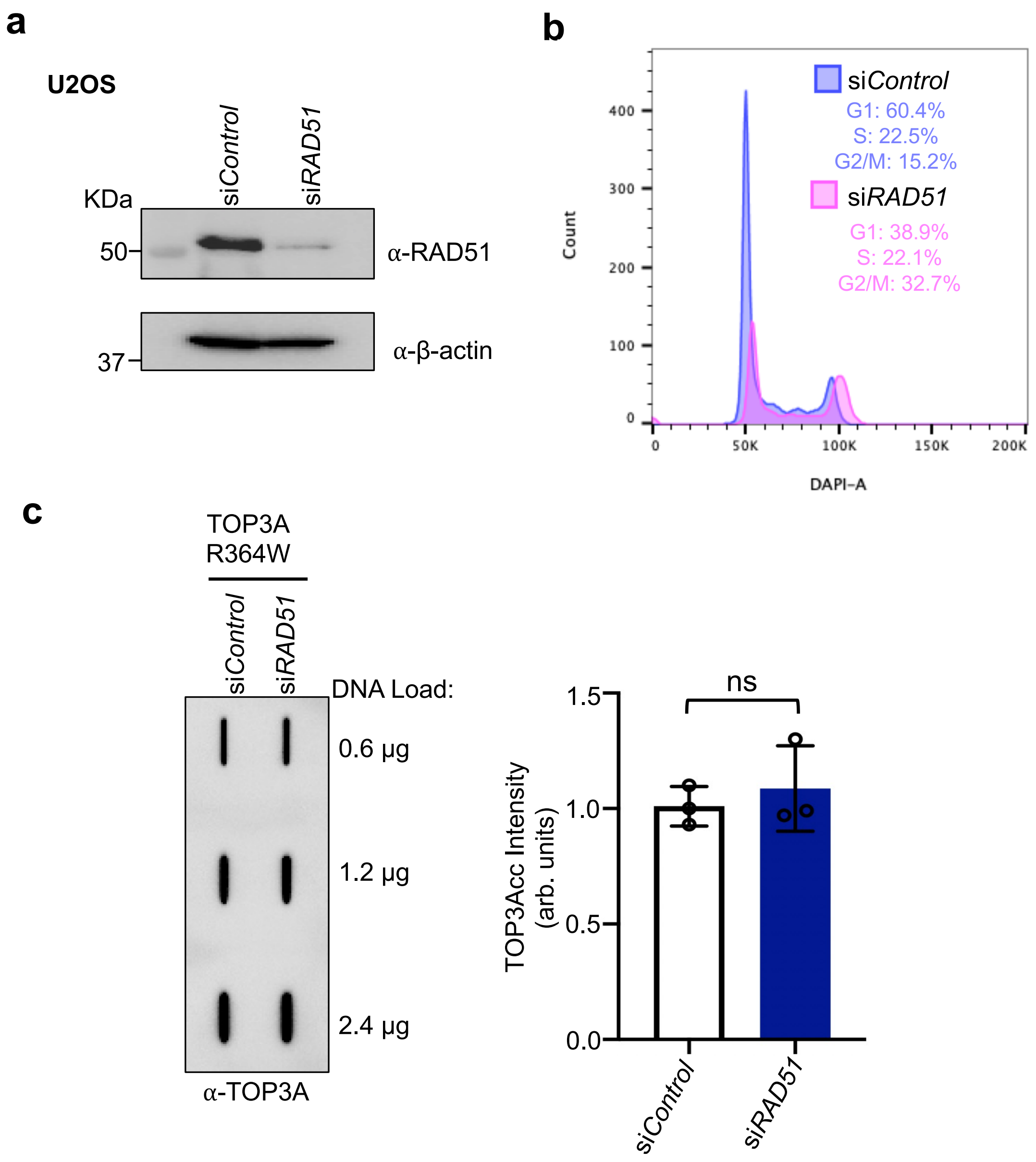

Supplementary Figure 3: Impact of RAD51 depletion on TOP3Accs in U2OS cells.

a Western blots of the expression level of RAD51 72 h after siRNA transfection. **b** Histogram showing the cell cycle distribution by fluorescence-activated cell sorting (FACS) analysis of the DNA content (DAPI) in RAD51-deficient (siRAD51) and control U2OS cells. **c** Left panel: HCT116 were transfected with either siControl or siRAD51 and then re-transfected with TOP3A-R364W to perform RADAR assay for TOP3Accs detection. Right panel: Quantitation of TOP3Accs from 3 independent RADAR assays as shown in left panel. Error bar indicates mean ± SD. P-values were obtained from two-tailed unpaired *t*-test with Welch's correction. ns=0.5639.

Supplementary Figure 4

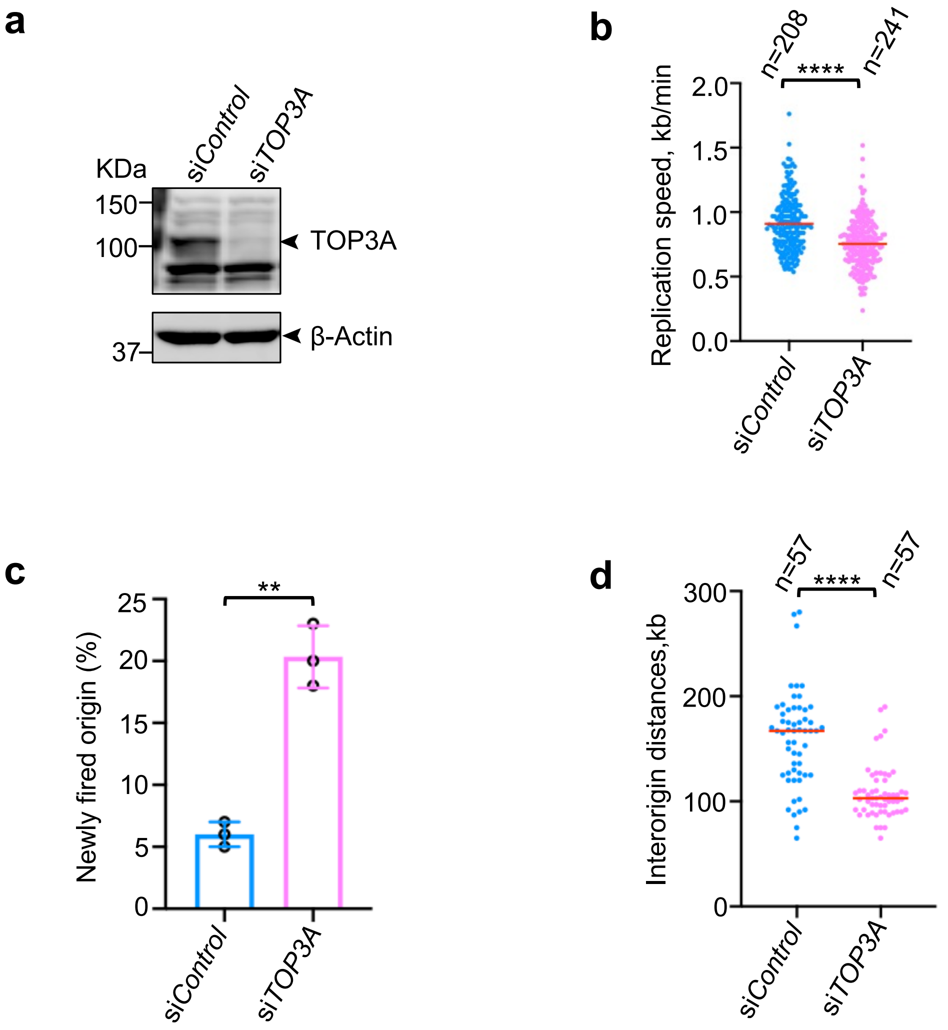

Supplementary Figure 4: Impact of TOP3A depletion on replication in U2OS cells.

a Western blots of the expression level of TOP3A 60 h after siRNA transfection. **b** Nascent DNA was labeled with CldU (30 min) followed by IdU (30 min) in U2OS cells as in Fig. **3a**. Dot plot showing the length of nascent DNA labeled by CldU in *wild-type* and TOP3A knockdown (siTOP3A) U2OS cells. The distribution of replication tract lengths is shown. Horizontal red lines indicate median values. n=the number of individual measures. Two-tailed Mann-Whitney test, ****p<0.0001. **c** Histogram showing the percentage of newly fired origins in siTOP3A and control cells. Percentage was calculated from the number of fibers with single green signals divided by the total number of fibers. Error bars indicate the mean value ± SD (n=3 independent experiments). Two-tailed unpaired *t*-test with Welch's correction, **p=0.0047. **d** Replication inter-origin distances in TOP3A knockdown and control cells. Horizontal red lines indicate median values. n=the number of individual measures. Two-tailed Mann-Whitney test, ****p<0.0001.

Supplementary Figure 5

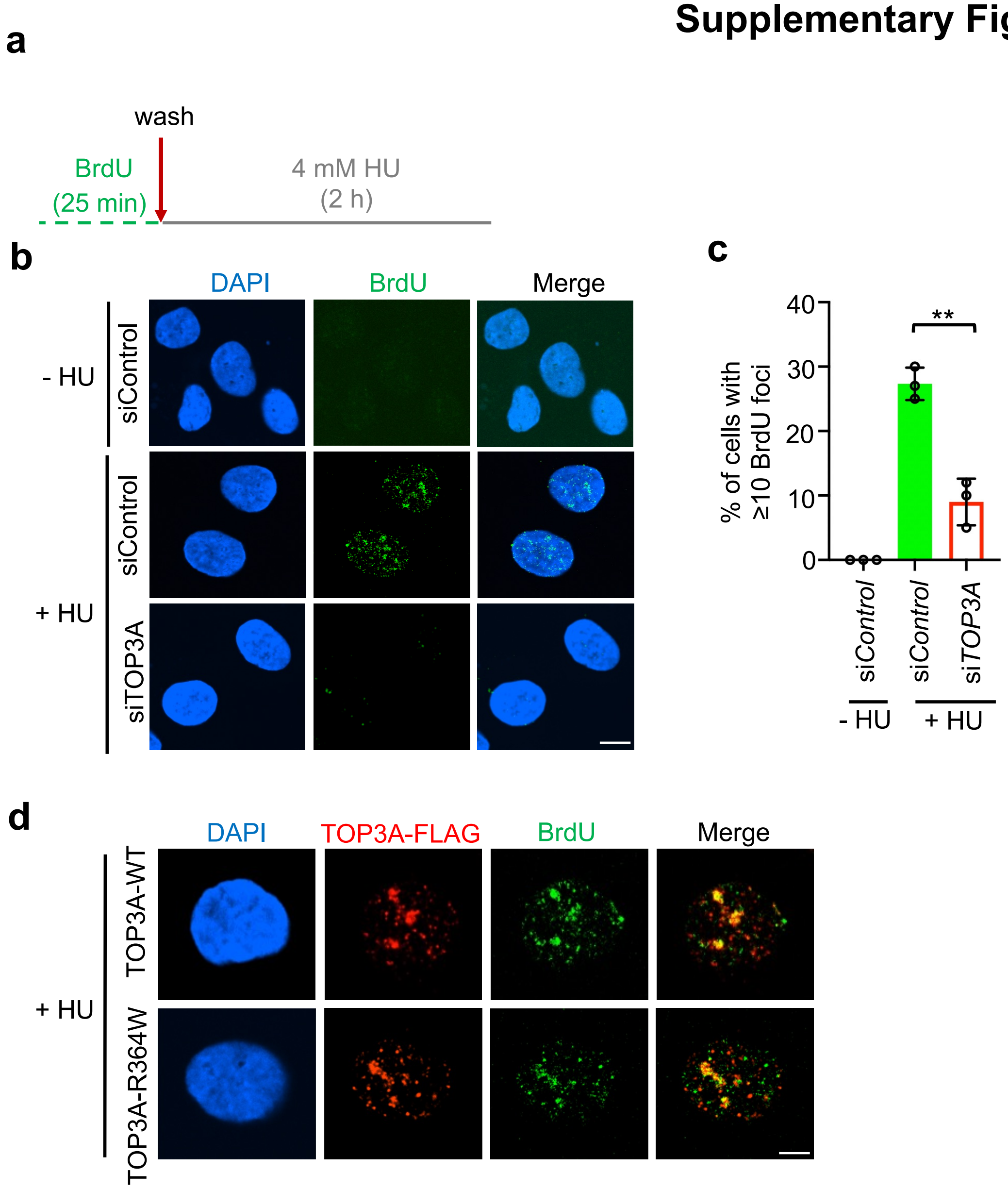

Supplementary Figure 5: Impact of TOP3A depletion at reversed fork upon replication stress.

a schematic representation of the native BrdU immunofluorescence assay for nascent ssDNA detection. **b** Representative images of BrdU foci-containing cells with and without HU treatment. Scale bar: 5μm. **c** Quantification of BrdU foci. Data represent mean ± SD from three independent experiments. Error bars indicate the mean value ± SD. Two-tailed unpaired *t*-test with Welch's correction, **p=0.0030. **d** Representative images showing the colocation of TOP3A with ssDNA (BrdU foci) in TOP3A-WT and TOP3A-R364W overexpressing cells. TOP3A foci were detected using ant-FLAG antibody. Scale bar: 10μm.

Supplementary Figure 6

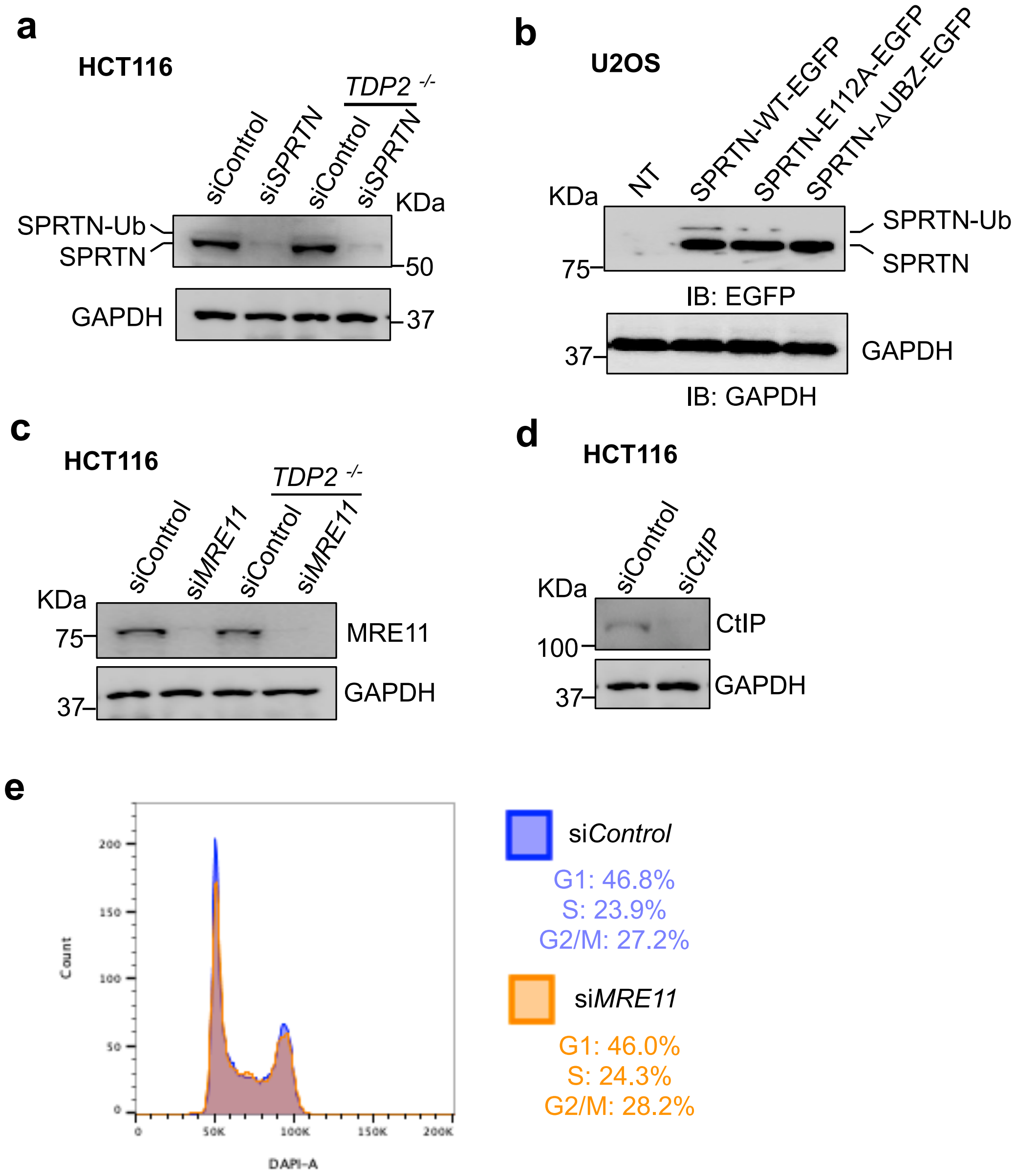

Supplementary Figure 6: Ectopic expression of SPRTN-EGFP plasmids and verification of depletion of SPRTN, TDP2 and MRE11.

a Western blot analysis of the expression of SPRTN) in indicated genotypes and cells. GAPDH was used as loading control. **b** Western blot of U2OS cells expressing EGFP-tagged SPRTN-WT, SPRTN-E112A (catalytic dead) and SPRTN-∆UBZ plasmids. Cells were transfected with the indicated FLAG-tagged SPRTN constructs for 48 h and subjected to Western blotting with α-EGFP antibody. GAPDH was used as loading control. **c–d** Western blot analysis of the expression of MRE11 (**c**), and CtIP (**d**) in indicated genotypes and cells. GAPDH was used as loading control. **e** Histogram showing the cell cycle distribution by fluorescence-activated cell sorting (FACS) analysis of the DNA content (DAPI) in MRE11-deficient (siMRE11) and control U2OS cells.

Supplementary Figure 7

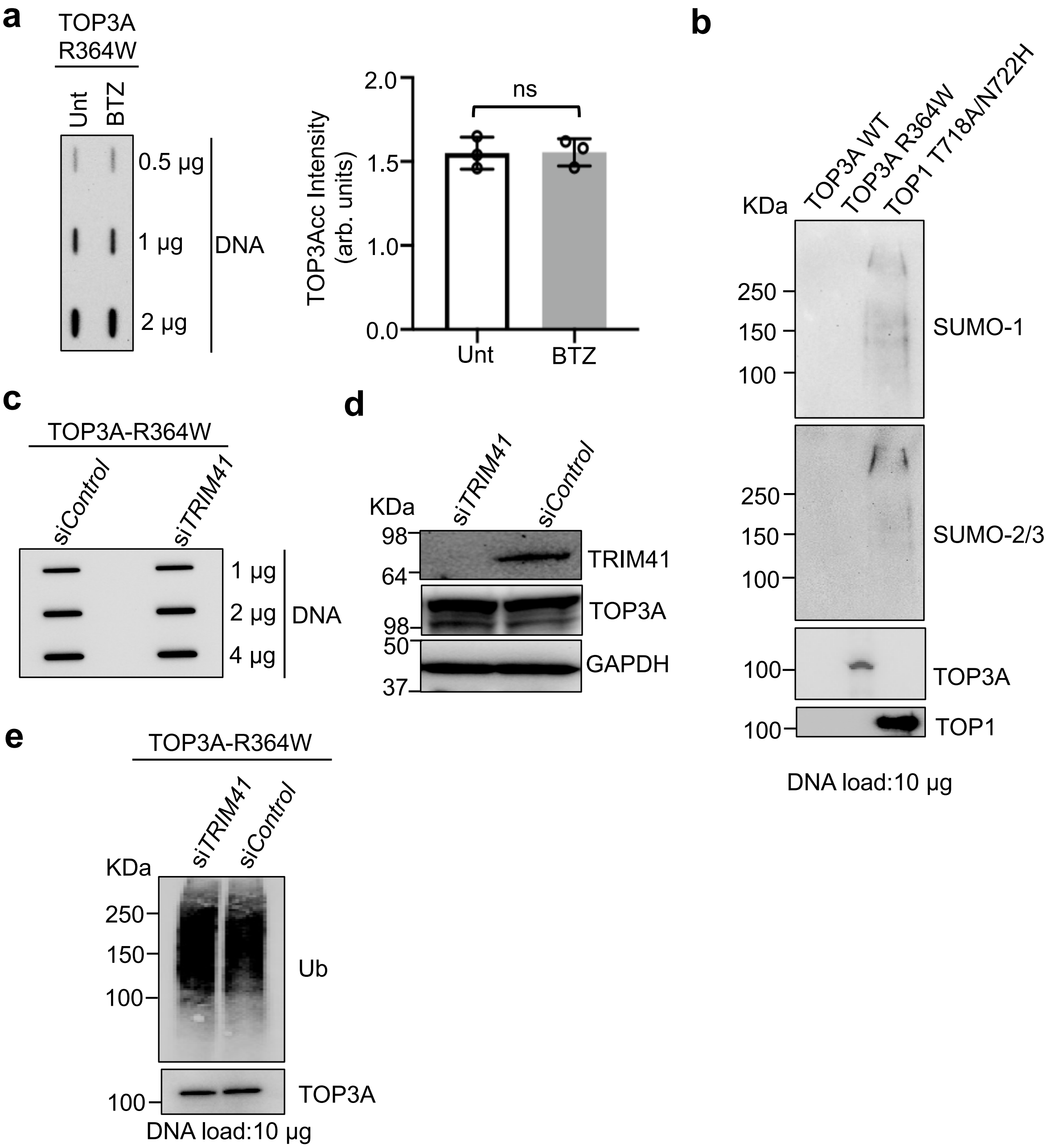

Supplementary Figure 7: Cellular TOP3Accs are not SUMOylated and degraded by proteasome.

a HCT116 cells were transfected with TOP3A-R364W for 48 h. Before harvest, cells were treated with proteasome inhibitor, bortezomib (1 μM, 2 h). TOP3Accs were detected by using anti-FLAG antibody. Quantification of TOP3Accs from three independent experiments as shown in left panel. Error bar indicates mean ± SD. Two-tailed unpaired *t*-test with Welch's correction, ns=0.9533. **b** HCT116 WT cells were transfected with indicated plasmids and analyzed by DUST assay as indicated. Transfection of TOP1 T118A/N122H plasmid was used as a control to detect SUMOylation. **c** Western blot analysis showing efficient knockdown of TRIM41 and TOP3A expression in indicated HCT116 cells. **d** HCT116 were transfected with siTRIM41 and then re-transfected with TOP3A-R364W to perform RADAR assay for TOP3Accs detection. **e** HCT116 cells were transfected with either siControl or siTRIM41, followed by TOP3A-R364W transfection, and analyzed by DUST assay as indicated.

Supplementary Figure 8

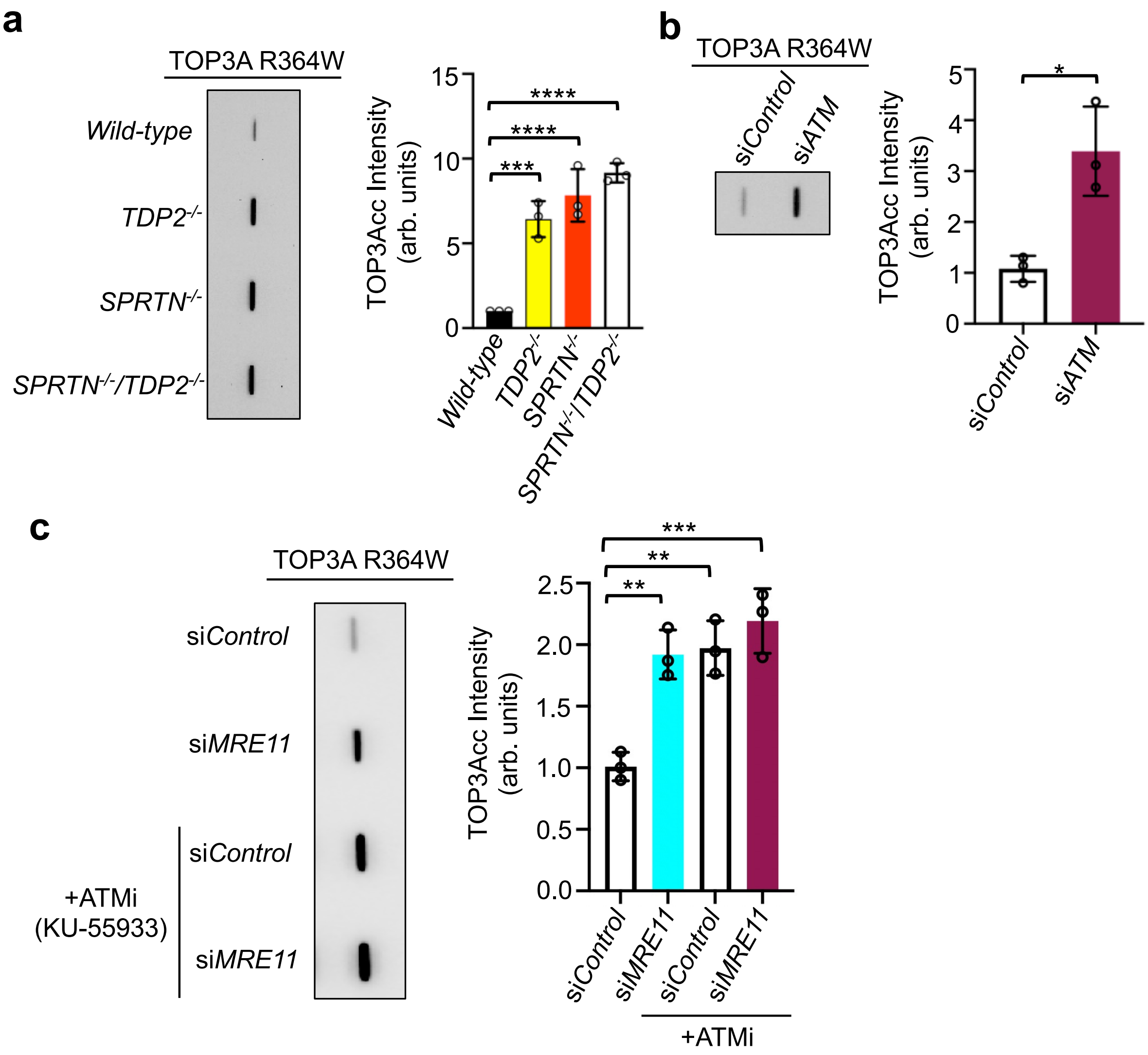

Supplementary Figure 8: Epistasis of SPRTN and TDP2 in TK6 cells, elevated TOP3Accs levels in ATM-depleted HCT116 cells, epistasis of MRE11 and ATM.

a Epistatic relationship between SPRTN and TDP2 in human TK6 cells. Left panel: representative slot-blot. WT, *SPRTN* KO, *TDP2 KO* and *SPRTN/TDP2* double-KO TK6 cells were transfected with TOP3A-R364W plasmid constructs for 48 h. TOP3Accs were detected by RADAR assay with anti-TOP3A antibody. Right panel: Quantitation of TOP3Accs from 3 independent RADAR assays as shown in left panel. Error bar indicates mean ± SD. Ordinary one-way ANOVA with Dunnett multiple comparison test. ***Adjusted p value=0.0004 (Wild-type vs *TDP2*$^{-/-}$), ****Adjusted p value ≤0.0001 (Wild-type vs *SPRTN*$^{-/-}$), ****Adjusted p value ≤0.0001 (Wild-type vs *SPRTN*$^{-/-}$/*TDP2*$^{-/-}$). **b** HCT116 WT cells were transfected with ATM siRNA and subsequently transfected with TOP3A-R364W plasmid and analyzed by RADAR assay for the detection of TOP3Accs. Right panel shows the quantification from three independent experiments as shown in left panel. Data are represented as mean± SD. Two-tailed unpaired *t*-test with Welch's correction, *p=0.0362. **c** Epistatic relationship between MRE11 and ATM in HCT116 cells. Left panel: representative slot blot of RADAR assay in either siControl or siMRE11 cells. Before harvest, cells were treated with either DMSO or the ATM inhibitor KU-55933 (20 μM) for 2 h. Right panel: quantitation of TOP3Accs from three independent experiments. Data are represented as mean± SD. Ordinary one-way ANOVA with Dunnett multiple comparison test. **Adjusted p value=0.0017 (Wild-type vs si*MRE11*), **Adjusted p value=0.0012 (Wild-type vs Wild-type+ATMi), ***Adjusted p value=0.0003 (Wild-type vs si*MRE11*+ATMi).

Supplementary Figure 9

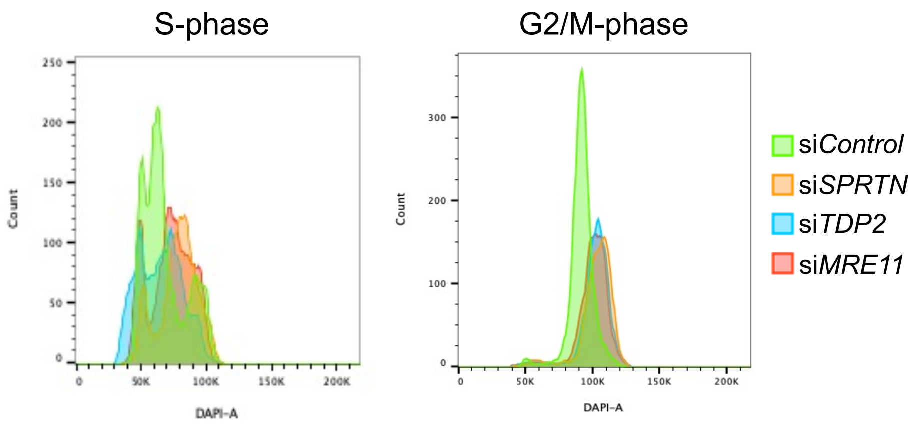

Supplementary Figure 9: Cell cycle phase-dependent expression of TOP3A-R364W-expressing cells.

TOP3A-R364W-expressing U2OS cells with indicated genotypes were synchronized in S and G2/M phases of the cell cycle with double-thymidine block. Histogram shows cell cycle distribution by fluorescence-activated cell sorting (FACS) analysis of cellular DNA content (DAPI).

Supplementary Figure 10

a

EdU and DAPI staining for cell cycle analyses:

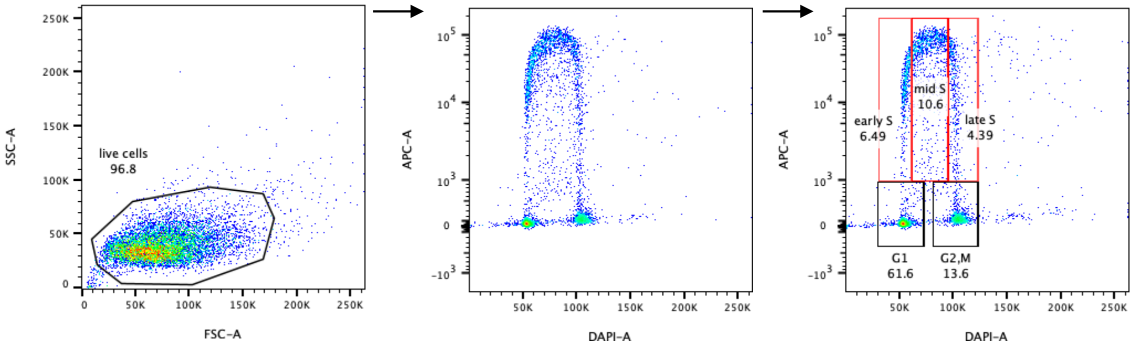

b

DAPI staining for cell cycle analyses:

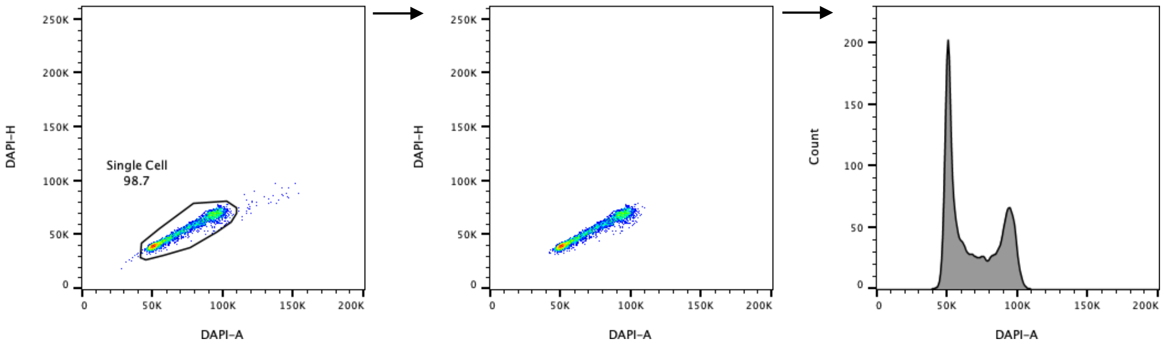

Supplementary Figure 10: Gating strategy for flow cytometry analysis.

a Live cell population were gated based on FSC/SSC; second round of gating was based on EdU/DAPI. **b** Single cell population were gated according to DAPI-H/DAPI-A.
