# Supplementary material for: Replication-associated formation and repair of human topoisomerase IIIα cleavage complexes
Source: Nat Commun. 2023 Apr 6;14:1925. doi: 10.1038/s41467-023-37498-6 (PMC10079683; doi:10.1038/s41467-023-37498-6)
Supplement: Supplementary file 3 — Reporting Summary [file 41467_2023_37498_MOESM3_ESM.pdf]

nature portfolio

Corresponding author(s): Yves Pommier

Last updated by author(s): Feb 21, 2023

# Reporting Summary

Nature Portfolio wishes to improve the reproducibility of the work that we publish. This form provides structure for consistency and transparency in reporting. For further information on Nature Portfolio policies, see our Editorial Policies and the Editorial Policy Checklist.

## Statistics

For all statistical analyses, confirm that the following items are present in the figure legend, table legend, main text, or Methods section.

| n/a | Confirmed | |
|---|---|---|
| ☐ | ☒ | The exact sample size (*n*) for each experimental group/condition, given as a discrete number and unit of measurement |
| ☐ | ☒ | A statement on whether measurements were taken from distinct samples or whether the same sample was measured repeatedly |
| ☐ | ☒ | The statistical test(s) used AND whether they are one- or two-sided<br>*Only common tests should be described solely by name; describe more complex techniques in the Methods section.* |
| ☒ | ☐ | A description of all covariates tested |
| ☒ | ☐ | A description of any assumptions or corrections, such as tests of normality and adjustment for multiple comparisons |
| ☐ | ☒ | A full description of the statistical parameters including central tendency (e.g. means) or other basic estimates (e.g. regression coefficient) AND variation (e.g. standard deviation) or associated estimates of uncertainty (e.g. confidence intervals) |
| ☐ | ☒ | For null hypothesis testing, the test statistic (e.g. *F*, *t*, *r*) with confidence intervals, effect sizes, degrees of freedom and *P* value noted<br>*Give P values as exact values whenever suitable.* |
| ☒ | ☐ | For Bayesian analysis, information on the choice of priors and Markov chain Monte Carlo settings |
| ☒ | ☐ | For hierarchical and complex designs, identification of the appropriate level for tests and full reporting of outcomes |
| ☒ | ☐ | Estimates of effect sizes (e.g. Cohen's *d*, Pearson's *r*), indicating how they were calculated |

*Our web collection on statistics for biologists contains articles on many of the points above.*

## Software and code

Policy information about availability of computer code

| | |
|---|---|
| Data collection | BioRad ChemiDoc MP Imaging System ; FiberVision Automated Scanner (Genomic Vision); Zeiss LSM 880 Airyscan confocal/super resolution microscope; Nikon SoRa super-resolution spinning disk microscope equipped with a Plan Fluor 60x oil objective lens and a camera (CoolSNAP HQ2; Photometrics), BD LSRFortessa cell analyzer with FACSDiva software (version 6.2). |
| Data analysis | Image Lab Software for PC version 6.1; ImageJ (Fiji, 2020) ; Graph Pad Prism 8.0 ; Flow Jo 10.8.1; FiberStudio software version 2.0 (Genomic Vision). |

For manuscripts utilizing custom algorithms or software that are central to the research but not yet described in published literature, software must be made available to editors and reviewers. We strongly encourage code deposition in a community repository (e.g. GitHub). See the Nature Portfolio guidelines for submitting code & software for further information.

# Data

Policy information about availability of data

All manuscripts must include a data availability statement. This statement should provide the following information, where applicable:

- Accession codes, unique identifiers, or web links for publicly available datasets
- A description of any restrictions on data availability
- For clinical datasets or third party data, please ensure that the statement adheres to our policy

Source data are provided with this paper. Source data are provided as a 'Source Data' file. All data related to this paper are available from the corresponding author upon reasonable request.

# Human research participants

Policy information about studies involving human research participants and Sex and Gender in Research.

| | |
|---|---|
| Reporting on sex and gender | N/A |
| Population characteristics | N/A |
| Recruitment | N/A |
| Ethics oversight | N/A |

Note that full information on the approval of the study protocol must also be provided in the manuscript.

# Field-specific reporting

Please select the one below that is the best fit for your research. If you are not sure, read the appropriate sections before making your selection.

☒ Life sciences ☐ Behavioural & social sciences ☐ Ecological, evolutionary & environmental sciences

For a reference copy of the document with all sections, see nature.com/documents/nr-reporting-summary-flat.pdf

# Life sciences study design

All studies must disclose on these points even when the disclosure is negative.

| | |
|---|---|
| Sample size | Sample size was determined to ensure statistical analyses. No statistical methods were used to predetermine sample size, which were chosen based on previous experience with these type of experiments . For DNA combing analysis and microscopy cell counting, we analyze 50 to 250 signals or cells that generated sufficient statistics for the effect sizes of interest. |
| Data exclusions | No data exclusions. |
| Replication | All the experiments performed in this study were repeated either 2 or 3 times as indicated in the figure legends. All attempts at replication were successful. |
| Randomization | The experiments were not randomized as this study was not related to drug efficacy and safety in animal models or human. We used cell lines only for all the experiments. Randomization is not generally used for other experiments except for DNA combing analysis, fibers were random selected by the FiberStudio software. For manually counting of IdU and CldU signals, images were randomly selected. |
| Blinding | The investigators were not blinded to allocation during experiment and outcome assessment. Blinding was not relevant for our study because analyses were analyst independent. |

# Reporting for specific materials, systems and methods

We require information from authors about some types of materials, experimental systems and methods used in many studies. Here, indicate whether each material, system or method listed is relevant to your study. If you are not sure if a list item applies to your research, read the appropriate section before selecting a response.

## Materials & experimental systems

| n/a | Involved in the study |
|---|---|
| ☐ | ☒ Antibodies |
| ☐ | ☒ Eukaryotic cell lines |
| ☒ | ☐ Palaeontology and archaeology |
| ☒ | ☐ Animals and other organisms |
| ☒ | ☐ Clinical data |
| ☒ | ☐ Dual use research of concern |

## Methods

| n/a | Involved in the study |
|---|---|
| ☒ | ☐ ChIP-seq |
| ☐ | ☒ Flow cytometry |
| ☒ | ☐ MRI-based neuroimaging |

# Antibodies

Antibodies used

Primary antibodies used in this study are as follows:

anti-TOP3A (dilution 1:1000, Proteintech, Rosemont, IL, Cat#:14525-1-AP)
anti-FLAG (dilution 1:1000, Sigma-Aldrich, Cat# F1804, Clone M2)
anti-GAPDH (dilution 1:2000, Cell Signaling Technology, Cat# 2118, Clone 14C10)
anti-PCNA (dilution 1:1000, Cell Signaling Technology, Cat# 13110, Clone D3H8P)
anti-CDC45 (dilution 1:100, Cell Signaling Technology, Cat# 11881S)
anti-H3 dilution 1:1000, Cell Signaling Technology, Cat# 9715)
anti-RPA1 (dilution 1:500, Cell Signaling Technology, Cat# 2267)
anti-RPA2 (dilution 1:500, Cell Signaling Technology, Cat# E8X5P)
anti-EGFP (dilution 1:500, Clontech, Cat# 632380, Clone JL-8)
anti-Ub (dilution 1:500, Cell Signaling Technology, Cat# 3936)
anti-SPRTN (dilution 1:1000, Atlas Antibodies, Cat# HPA025073)
anti-MRE11 (dilution 1:1000, GeneTex, Cat# GTX70212, Clone 12D7)
anti-CtIP (dilution 1:1000, Cell Signaling Technology, Cat# 9201, Clone D76F7)
anti-b-actin (dilution 1:3000, Sigma-Aldrich, Cat# A5411)
anti-BrdU for IdU (dilution 1:20, BD Biosciences, Cat# 347580)
anti-BrdU for CldU (dilution 1:100, Abcam, Cat# Ab6326)
anti-single strand DNA (dilution 1:200, Millipore, Cat# MAB3034)
anti-TOMM20 (1:500 dilution, Sigma, Cat# HPA011562)
anti-SUMO-1 (dilution 1:1000, Cell Signaling Technology, Cat# 4940)
anti-SUMO-2/3 (dilution 1:1000, Cell Signaling Technology, Cat# 4971)
anti-TRIM41 (dilution 1:1000, Abcam, Cat# ab111580)
anti-BLM (dilution 1:1000, Santa Cruz Biotechnology, Cat# sc-365753)
anti-RMI1(dilution 1:1000, Thermo Fischer Scientific, Cat# 14630-1-AP)
anti-pATR(T1989) (dilution 1:1000, Abcam, Cat# ab223258
anti-pChk1(S345) (dilution 1:1000, Cell Signaling Technology, Cat# 2348, Clone 133D3)
anti-pATM(S1981) (dilution 1:1000, Cell Signaling Technology, Cat# 13050, Clone D25E5)
anti-pChk2(Thr68) (dilution 1:1000, Cell Signaling Technology, Cat# 2661)
anti-gH2AX(S139) (dilution 1:500, Millipore, Cat# 05-636, Clone JBW301)
anti-RAD51 (dilution 1:350, Sigma-Aldrich, Cat# PC130)
anti-TOP1 (dilution 1:1000, BD Biosciences, Cat# 556597)
anti-TOP2A (dilution 1:1000, Millipore, Cat# MAB4197)
anti-TOP2B (dilution 1:1000, BD Biosciences, Cat# 611493)

The following secondary antibodies were used in this study:

anti-mouse IgG ECL, HRP conjugated (dilution 1:4000, GE Healthcare, Cat# NA9310)
anti-rabbit IgG ECL, HRP conjugated (dilution 1:4000, GE Healthcare, Cat# NA9340)
anti-mouse Alexa Fluor 488 (dilution 1:1000, Thermo Fischer Scientific, Cat# A28175)
anti-rabbit Alexa Fluor 568 (dilution 1:1000, Thermo Fischer Scientific, Cat# A-11011)
anti-mouse Cy3 (1:100 dilution, Abcam, Cat# AB97035)
anti-rat Cy5 (1:100 dilution, Abcam, Cat# AB6565)
anti-mouse BV480 for ssDNA (1:50 dilution, Jackson ImmunoResearch, Cat#115-685-166)

Validation

No in-house antibodies were used in this study. All commercial antibodies were validated by suppliers and previous references. Antibodies were further validated by siRNA transfection, as indicated below:

anti-TOP3A: https://www.ptglab.com/products/TOP3A-Antibody-14525-1-AP.htm
anti-FLAG: https://www.sigmaaldrich.com/US/en/product/sigma/f1804
anti-GAPDH: https://www.cellsignal.com/products/primary-antibodies/gapdh-14c10-rabbit-mab/2118
anti-PCNA: https://www.cellsignal.com/products/primary-antibodies/pcna-d3h8p-xp-rabbit-mab/13110
anti-CDC45: https://www.cellsignal.com/products/primary-antibodies/cdc45-d7g6-rabbit-mab/11881
anti-H3: https://www.cellsignal.com/products/primary-antibodies/histone-h3-antibody/9715
ant-RPA1: https://www.cellsignal.com/products/primary-antibodies/rpa70-rpa1-antibody/2267
anti-RPA2: https://www.cellsignal.com/products/primary-antibodies/rpa32-rpa2-e8x5p-xp-rabbit-mab/35869
anti-EGFP: https://www.alzforum.org/antibodies/gfp-jl-8

anti-Ub: https://www.cellsignal.com/products/primary-antibodies/ubiquitin-p4d1-mouse-mab/3936
ant-SPRTN: https://www.atlasantibodies.com/products/antibodies/primary-antibodies/triple-a-polyclonals/sprtn-antibody-hpa025073/
anti-MRE11: https://www.genetex.com/Product/Detail/Mre11-antibody-12D7/GTX70212
anti-CtIP: https://www.cellsignal.com/products/primary-antibodies/ctip-d76f7-rabbit-mab/9201
anti- -actin: https://www.sigmaaldrich.com/US/en/product/sigma/a5441
anti-BrdU for IdU:https://www.bdbiosciences.com/en-us/products/reagents/flow-cytometry-reagents/clinical-discovery-research/single-color-antibodies-ruo-gmp/purified-mouse-anti-brdu.347580
anti-BrdU for CldU:https://www.abcam.com/brdu-antibody-bu175-icr1-proliferation-marker-ab6326.html
anti-single strand DNA :https://www.emdmillipore.com/US/en/product/Anti-DNA-Antibody-single-stranded-clone-16-19,MM_NF-MAB3034
anti-TOMM20: https://www.sigmaaldrich.com/US/en/product/sigma/hpa011562
anti-SUMO-1 : https://www.cellsignal.com/products/primary-antibodies/sumo-1-c9h1-rabbit-mab/4940
anti-SUMO-2/3: https://www.cellsignal.com/products/primary-antibodies/sumo-2-3-18h8-rabbit-mab/4971
anti-TRIM41: https://www.abcam.com/trim41-antibody-ab111580.html
anti-BLM: https://www.scbt.com/p/blm-antibody-b-4
anti-RMI1: https://www.thermofisher.com/antibody/product/RMI1-Antibody-Polyclonal/14630-1-AP
anti-pATR(T1989) :https://www.abcam.com/atr-phospho-t1989-antibody-epr21991-ab223258.html
anti-pChk1(S345) :https://www.cellsignal.com/products/primary-antibodies/phospho-chk1-ser345-133d3-rabbit-mab/2348
anti-pATM(S1981): https://www.cellsignal.com/products/primary-antibodies/phospho-atm-ser1981-d25e5-rabbit-mab/13050
anti-pChk2(Thr68): https://www.cellsignal.com/products/primary-antibodies/phospho-chk2-thr68-antibody/2661:
anti-gH2AX(S139): https://www.emdmillipore.com/US/en/product/Anti-phospho-Histone-H2A.X-Ser139-Antibody-clone-JBW301,MM_NF-05-636

anti-RAD51: https://www.sigmaaldrich.com/US/en/product/mm/pc130

anti-TOP1: https://www.citeab.com/antibodies/2414002-556597-bd-pharmingen-purified-mouse-anti-human-dna topoisomerase I

anti-TOP2A: https://www.emdmillipore.com/US/en/product/Anti-Topoisomerase-II-Antibody-clone-KiS1,MM_NF-MAB4197

anti-TOP2B: https://www.bdbiosciences.com/en-us/products/reagents/microscopy-imaging-reagents/immunofluorescence-reagents/purified-mouse-anti-human-topo-ii.611493

## Eukaryotic cell lines

Policy information about cell lines and Sex and Gender in Research

| | |
|---|---|
| Cell line source(s) | HEK293,HCT116 and U2OS cells were obtained from the NCI Developmental Therapeutics Program.TK6 cells were obtained from Department of Radiation Genetics, Kyoto University, Japan. Original source of TK6: Japanese Collection of Research Bio resources (JCRB, Japan). |
| Authentication | Cell line authentication was carried out using short tandem repeat analysis at Frederick National Laboratory. |
| Mycoplasma contamination | Cells were routinely tested for mycoplasma by MicoAlert (Lonza) and found negative. |
| Commonly misidentified lines (See ICLAC register) | No commonly misidentified cell lines was used. |

## Flow Cytometry

### Plots

Confirm that:

☒ The axis labels state the marker and fluorochrome used (e.g. CD4-FITC).

☒ The axis scales are clearly visible. Include numbers along axes only for bottom left plot of group (a 'group' is an analysis of identical markers).

☒ All plots are contour plots with outliers or pseudocolor plots.

☒ A numerical value for number of cells or percentage (with statistics) is provided.

### Methodology

| | |
|---|---|
| Sample preparation | Asychronous and/or synchronized cells were harvested after EdU pulse labelling and then performed ClickiT reactions with kit with click chemistry (for EdU and DAPI staining) and/or without click chemistry (for DAPI staing only). |
| Instrument | BD LSRFortessa cell analyzer with FACSDiva software (version 6.2) |
| Software | Flow Jo 10.8.1 |
| Cell population abundance | ~5,000-10,000 cell populations |

Gating strategy

Live cell population were gated based on FSC/SSC; second round of gating was based on EdU/DAPI, as shown in Supplementary Fig. 10a.
Single cell population gated according to DAPI-H/DAPI-A were analyzed as the gates shown in Supplementary Fig. 10b.

☒ Tick this box to confirm that a figure exemplifying the gating strategy is provided in the Supplementary Information.
